# Optimization of Preparation of Antioxidative Peptides from Pumpkin Seeds Using Response Surface Method

**DOI:** 10.1371/journal.pone.0092335

**Published:** 2014-03-17

**Authors:** Sanhong Fan, Yanan Hu, Chen Li, Yanrong Liu

**Affiliations:** College of Life Science, Shanxi University, Taiyuan, China; CNR, Italy

## Abstract

Protein isolates of pumpkin (*Cucurbita pepo* L) seeds were hydrolyzed by acid protease to prepare antioxidative peptides. The hydrolysis conditions were optimized through Box-Behnken experimental design combined with response surface method (RSM). The second-order model, developed for the DPPH radical scavenging activity of pumpkin seed hydrolysates, showed good fit with the experiment data with a high value of coefficient of determination (0.9918). The optimal hydrolysis conditions were determined as follows: hydrolyzing temperature 50°C, pH 2.5, enzyme amount 6000 U/g, substrate concentration 0.05 g/ml and hydrolyzing time 5 h. Under the above conditions, the scavenging activity of DPPH radical was as high as 92.82%.

## Introduction

With the improvement of people’s living standard, many kinds of diseases caused by reactive oxygen species (ROS) and free radicals are bringing serious damage to human health [1], [2]. In recent years, preparation of antioxidative compounds has become a research hotspot in biology, medicine and food science [3], [4]. Nowadays, more and more attention has been focused on plant protein hydrolysates with antioxidative properties [5], [6], which are considered as potential natural substitutes of artificial antioxidants (BHA, BHT, TBHQ), as well as exogenous antioxidants in human nutrition [7].

Considering the nutritional quality and cost, many legumes, oilseeds and their by-products of the oil industry are suitable as protein sources to produce protein hydrolysates for food or non-food application. Some materials have been extensively studied including soybean protein [8], peanut protein [9], [10], cottonseed protein [11], etc. Many studies show that the antioxidative activity of individual peptide depends on amino acids in sequence and molecular mass [12]–[14]. However, the hydrolysate with antioxidative properties of one kind of protein may also be affected by some other factors, such as pH, temperature, the enzyme amount and hydrolysis time. When many different factors need to be considered, the response surface method (RSM) is an effective tool for optimization of conditions [15], [16].

Pumpkin seed is one of the most important oilseeds in many countries (India, China, etc.), which is also a popular snack. Nearly 90% of the total output in these countries is used for extraction of oil [17], leaving a large amount of residue which contains 60%–65% of high value protein. Although some defatted flour is utilized increasingly, a huge amount of the residue is still being discarded causing waste and pollution. Additionally, pumpkin seeds could also be used as raw material for production of high quality protein products which are nutrition supplements for food formulations, as well as functional agents [18]–[21].

Most notably, pumpkin seed is a valuable raw material for preparation of antioxidative peptides owing to its high protein content, availability and low cost. In this paper, antioxidative peptides were prepared by hydrolysis from pumpkin seed. The effects of enzyme amount, substrate concentration and hydrolysis time on the DPPH radical scavenging ability of pumpkin seed hydrolysates were investigated. Further more, the optimal hydrolysis conditions were established through RSM.

## Materials and Methods

### Materials

Pumpkin seeds (*Cucurbita pepo* L.) were provided by “Jinlilai” Co., Shanxi, China. Enzyme was acid protease with a declared activity of 100,000 U/g purchased from Imperial Jade Bio-technology Co., China. 1,1-diphenyl-2-picrylhydrazyl (DPPH) was obtained from Sigma Chemicals Co. All the other chemicals are chemically pure (chemical reagent).

### Preparation of Protein Isolate

The protein isolate was prepared in alkaline solution followed by isoelectric precipitation, as described by Sathe [22] with some modifications. Pumpkin seeds were dehulled to get kernels which were ground in a hammer mill. The ground kernels were then defatted by supercritical carbon dioxide to give the defatted flour, which was allowed to mix with water in the ratio of 1/30 (w/v). The pH of the mixed solution was adjusted to pH 11 by 1 N sodium hydroxide (NaOH). The mixed solution was stirred at 50°C for 1.5 h, centrifuged at 5000 r/min for 20 min. The supernatant was collected and adjusted to pH 5.3 by 1 N hydrochloric acid (HCl). Then the generated pH 5.3 solution was centrifuged at 5000 r/min for 20 min. The supernatant was discarded and the precipitate was collected and dried using a freeze drier.

### Experimental Design

In preliminary studies, amount of enzyme, substrate concentration and time were found to have a significant influence on the degree of hydrolysis (DH) and DPPH radical-scavenging activity. The effect of three independent variables on the scavenging activity and their optimum levels were investigated by RSM, using Box-Behnken experimental design. Here, 15 experiments were carried out (with three replicates at the centre of the design). The range and central point values of the independent variables (Table 1) were assorted based on the results of preliminary experiments. The full experimental design with respect to predicated values and experimental values for the response (DPPH radical-scavenging activity) is presented in Table 2.

**Table 1:** Factors and levels in the response surface design.

Factor	Symbol	Levels		
		−1	0	1
Enzyme amount (U/g)	x_1_	2000	5000	8000
Substrate concentration (g/ml)	x_2_	0.02	0.04	0.06
Time (h)	x_3_	2	4	6

**Table 2:** Box-Behnken design matrix and the response values for the DPPH free radical scavenging ability (%) of pumpkin seed hydrolysates.

Treat	Variable levels			DPPH free radical scavenging ability (%)	
	*x_1_*	*x_2_*	*x_3_*	Experimental (Y_0_)	Predicted (Y_i_)
1	0	−1	−1	80.05	79.8937
2	0	−1	1	84.47	85.0288
3	0	0	0	91.32	90.9767
4	0	0	0	90.95	90.9767
5	0	1	−1	90.84	90.2813
6	0	0	0	90.66	90.9767
7	−1	−1	0	81.63	81.6350
8	1	−1	0	84.72	84.3125
9	1	0	1	91.51	91.3588
10	−1	1	0	90.78	91.1875
11	1	1	0	92.56	92.5550
12	−1	0	1	90.74	90.1763
13	0	1	1	92.28	92.4363
14	1	0	−1	87.99	88.5538
15	−1	0	−1	85.54	85.6913

### Hydrolysis of Protein Isolates

The enzymatic hydrolysis was carried out as stated in Table 1, according to the experimental plan in Table 2. The hydrolysis was performed in a glass vessel under controlled conditions. The mixture was incubated at 50°C and pH 2.5. Then the mixture was separated to 3 portions, which are allowed to heat up to 100°C in 5 min and react for 2 h, 4 h and 6 h, respectively.

The mixture with inactivated enzyme was centrifuged at 5000 r/min for 10 min and the supernatant was used for further analysis.

### Determination of the Degree of Hydrolysis

The degree of hydrolysis was determined according to the method of Chalamaiah, M. et al. [23]. An aliquot (10 ml) was taken out from the supernatant of protein hydrolysis solution, then mixed with 10 ml of 20% trichloroacetic acid (TCA) and centrifuged at 5000 rpm for 30 min at room temperature. The supernatant was decanted and nitrogen analysis was carried out with the micro-Kjeldahl method [24]. The degree of hydrolysis (%) can be calculated according to equation (1).



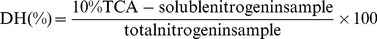
(1)


### Scavenging Activity of DPPH Radical

The scavenging activity of DPPH radical was measured according to the method of Shimada [25] with some modifications. In each test, an aliquot (2 ml) of sample solution with identical concentration was allowed to mix with 2 ml of DPPH (0.2 mmol/L in ethanol). The reaction mixture was incubated for 50 min in dark at room temperature. Absorbance of the resulting solution at 517 nm was measured by UV-Vis spectrometer (UV-2800AH, Shanghai, China). Ethanol was used to controlling solvent. The radical-scavenging capacity of samples was evaluated by increasing/decreasing the concentration of DPPH radical calculated by equation (2).



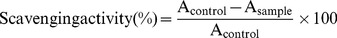
(2)


### Statistical Analysis

Data from the Box-Behnken experimental design (Table 2) was analyzed to determine the optimum combination of variables. The polynomial model proposed is given as equation (3):



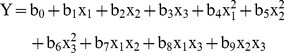
(3)where Y is the predicted response variable (scavenging capacity of DPPH radical), b_0_ is the regression coefficient of intercept term, b_1_, b_2_, b_3_ are linear regression coefficients, b_4_, b_5_, b_6_ are squared regression coefficients and b_7_, b_8_, b_9_ are interaction regression coefficients.

The significance of each coefficient of the resulted model was determined by using the Student t-test and p-value. The proportion of the variance expressed by the models is obtained by determination of multiple coefficient R^2^. Finally, the fitted polynomial equation was presented as surface plots in order to visualize the relationship between the response and experimental levels of each factor and search out the optimum conditions. The analysis software used for this study was Minitab 15. (Minitab Inc.).

## Results and Discussion

### Model Fitting

RSM was applied to obtain the regression equation, which represents an empirical relationship between response (scavenging activity of DPPH radical) and the test variables (enzyme amount, substrate concentration and hydrolysis time), as given in the equation (4).



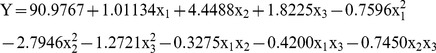
(4)


The significance of each coefficient was determined by using the *t*-values and P-values shown in Table 3. It can be seen that the largest effect was caused by the linear term including enzyme amount (x_1_), substrate concentration (x_2_) and time (x_3_), followed by the quadratic term of substrate concentration (x_2_^2^) and the quadratic term of time (x_3_^2^). The interaction terms did not have significant influence (P>0.05), which means that the interaction between the different factors did not influence the response.

**Table 3:** Significant test for each regression coefficient of the fitted regression model.

Variables	Regression coefficient	Standard error	*t-*value	P*-*value	Significance level
Constant	90.9767	0.3564	255.244	<0.0001	**
x_1_	1.0113	0.2183	4.633	0.006	**
x_2_	4.4488	0.2183	20.382	<0.0001	**
x_3_	1.8225	0.2183	8.350	<0.0001	**
x_1_^2^	−0.7596	0.3213	−2.364	0.064	
x_2_^2^	−2.7946	0.3213	−8.698	<0.0001	**
x_3_^2^	−1.2721	0.3213	−3.959	0.011	*
x_1_ x_2_	−0.3275	0.3087	−1.061	0.337	
x_1_ x_3_	−0.4200	0.3087	−1.361	0.232	
x_2_ x_3_	−0.7450	0.3087	−2.414	0.061	

*P<0.05, **P<0.01.

The calculated values by using the regression model were allowed to do a comparison with experimental values. The coefficient of determination R^2^ of calculated values was 0.9918. This indicates that the fitted model could explain 99.18% of the total variability within the range of values studied. The analysis of variance (Table 4) showed P-value was less than 0.01, which implied that the model itself is extremely significant. Meanwhile, the statistical analysis data revealed that linear and quadratic terms were extremely significant (P<0.01) while the interaction coefficients not (P>0.05). The lack of fit was also non-significant (P>0.05), which further validated the model.

**Table 4:** Analysis of variances for the fitted regression model.

Source	df	Sum of squares	Mean squares	F-value	P-value	Significance level
Regression	9	230.247	25.5830	67.12	<0.0001	**
Linear	3	193.084	64.3614	168.87	<0.0001	**
Square	3	33.808	11.2694	29.57	0.001	**
Interaction	3	3.355	1.1182	22.9	0.138	
Lack of fit	3	1.687	0.5623	5.14	0.167	
Pure error	2	0.219	0.1094			
Total	14	232.152			R^2^ = 0.9918	

*P<0.05, **P<0.01.

### Optimization of the Process

**Figure 1:**
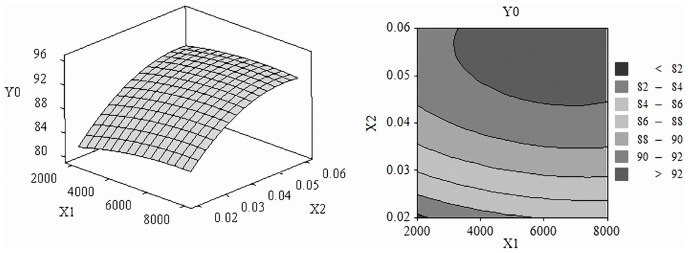
Effect of enzyme amount and substrate concentration. Response surface plots (A) and contour plots (B) of the DPPH radical scavenging activity of pumpkin seed hydrolysates affected by enzyme amount and substrate concentration.

The three-dimensional response surface graphs and contour maps were shown in Fig. 1 to Fig. 3, which illustrate the interactive effects of the independent variables on the scavenging capacity of DPPH radical. The shape of contour reflects the strength of the interaction effects, circular means interaction is not significant, while oval is opposite.

**Figure 2:**
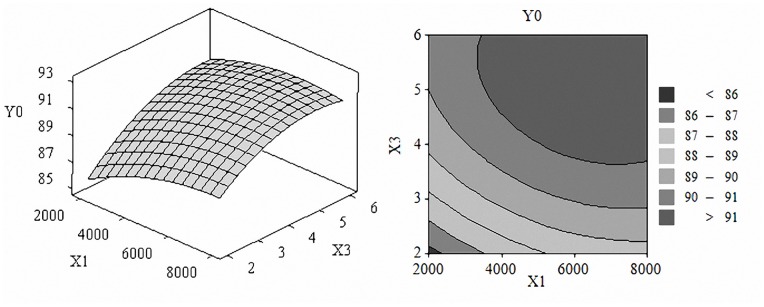
Effect of enzyme amount and hydrolysis time. Response surface plots (A) and contour plots (B) of the DPPH radical scavenging activity of pumpkin seed hydrolysates affected by enzyme amount and hydrolysis time.

**Figure 3:**
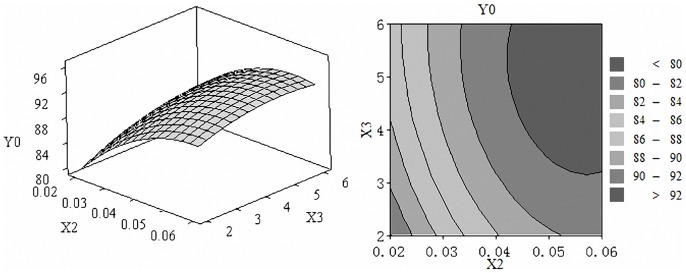
Effect of substrate concentration and hydrolysis time. Response surface plots (A) and contour plots (B) of the DPPH radical scavenging activity of pumpkin seed hydrolysates affected by substrate concentration and hydrolysis time.

Fig. 1 showed the effect of enzyme amount and substrate concentration on the scavenging capacity of DPPH radical, we learn that substrate concentration had greater influence on the response. Higher values of scavenging capacity were observed when the enzyme amount was 4000 to 7000 (U/g) and the substrate concentration was 0.04 to 0.06 (g/ml). Fig. 2 showed the effect of enzyme amount and time. Scavenging capacity of DPPH radical boosted with the increase of enzyme amount and time. Fig. 3 demonstrated the effect of substrate concentration and time. When substrate concentration was 0.04 to 0.06 g/ml and time was 3 to 6 h scavenging capacity of DPPH radical exhibited higher values of 92% to 96%.

These results implied that the response surface had a maximum point in the experimental range of the independent variables. The precise coordinates of optimum (i.e the values of three independent variables) were obtained by analytical procedure: enzyme amount 6181.82 U/g, substrate concentration 0.0543 g/ml, time 4.87 h. Thus, we can learn that the maximum predicted scavenging capacity under the optimal condition was 93.17%.

### Confirdmatory Tests

To confirm the validity of the suggested mathematical model, the trial experiments were conducted under the predicted optimal conditions. Take convenience into account, the actual optimum experimental parameters were modified as follows: enzyme amount 6000 U/g, substrate concentration 0.05 g/ml, time 5 h, with the temperature of 50°C and pH 2.5. Three parallel experiments were performed, and the values of DPPH radical scavenging activity were 92.15%, 93.08% and 93.23%, respectively. The mean of three replicate determinations was 92. 82%. The experimental value was in high agreement with the predicted value, which showed the validity of this response model.

## Conclusions and Discussion

To conclude, we carried out hydrolysis experiment for pumpkin seed protein isolate under 15 conditions by different combinations of enzyme amount, substrate concentration and hydrolysis time (independent variables). The second-order model developed for the DPPH radical scavenging activity of pumpkin seed hydrolysates was extremely significant (P<0.01) with a high value of coefficient of determination (0.9918). The surface and contour graphs indicated that maximum scavenging capacity of DPPH radical was 92.82%, which obtained under optimum hydrolysis condition (enzyme amount 6000 U/g, substrate concentration 0.05 g/ml, hydrolysis time 5 h, t temperature 50°C and pH 2.5), which could be validated by experiment. Thus, we believe that the optimum hydrolysis conditions can be obtained by RSM precisely.
